# Pharmacophore generation and atom-based 3D-QSAR of *N*-iso-propyl pyrrole-based derivatives as HMG-CoA reductase inhibitors

**DOI:** 10.1186/2191-2858-2-25

**Published:** 2012-07-02

**Authors:** Mahesh Kumar Teli, Rajanikant G K

**Affiliations:** 1 School of Biotechnology, National Institute of Technology Calicut, Calicut, 673601, Kerala, India

**Keywords:** HMG-CoA reductase, Hypercholesterolemia, *N*-iso-propyl pyrrole-based derivatives, Pharmacophore, 3D-QSAR

## Abstract

**Background:**

Coronary heart disease continues to be the leading cause of mortality and a significant cause of morbidity and account for nearly 30% of all deaths each year worldwide. High levels of cholesterol are an important risk factor for coronary heart disease. The blockage of 3-hydroxy-3-methylglutaryl-coenzyme A (HMG-CoA) reductase activity by small molecule inhibitors has been shown to inhibit hypercholesterolemia. Herein, we describe the development of effective and robust pharmacophore model and the structure–activity relationship studies of 43N-iso-propyl pyrrole-based derivatives previously reported for HMG-CoA reductase inhibition.

**Results:**

A 5-point pharmacophore model was developed and the generated pharmacophore model was used to derive a predictive atom-based 3D quantitative structure–activity relationship analysis (3D-QSAR) model for the studied dataset. The obtained 3D-QSAR model has an excellent correlation coefficient value (*r*^2^ = 0.96) along with good statistical significance as shown by high Fisher ratio (*F* = 143.2). The model also exhibited good predictive power confirmed by the high value of cross validated correlation coefficient (*q*^2^ = 0.672). Further, pharmacophoric model was employed for virtual screening to identify four potential HMG-CoA reductase inhibitors.

**Conclusions:**

The QSAR model suggests that electron-withdrawing character is crucial for the HMG-CoA reductase inhibitory activity. In addition to the electron-withdrawing character, hydrogen bond--donating groups, hydrophobic and negative ionic groups positively contribute to the HMG-CoA reductase inhibition. These findings provide a set of guidelines for designing compounds with better HMG-CoA reductase inhibitory potential.

## Background

High levels of cholesterol are linked to atherosclerosis, which is the accumulation of cholesterol-rich fatty deposits in arteries. This can cause arteries to narrow or become blocked, slowing or stopping the flow of blood to vital organs, especially the heart, brain and kidney [1-5]. When atherosclerosis blocks arteries that supply blood to the brain, it can cause an ischemic stroke. Atherosclerotic heart disease is the leading cause of death, accounting for one-third of all deaths and atherosclerotic interference with blood supply to the brain (ischemic stroke) is the third most common cause of death after cancer [6-9]. The biosynthetic pathway for cholesterol involves more than 25 different enzymes and the major rate-limiting step in this pathway is regulated by the 3-hydroxy- 3-methylglutaryl Coenzyme A (HMG-CoA) reductase. Inhibition of HMG-CoA reductase leads to a decrease in intrahepatic cholesterol concentration [10-14].

In recent years, several successful strategies for the inhibition of cholesterol biosynthesis have effectively been demonstrated in preclinical and clinical settings. For examples, ezetimibe and statins have been approved for the treatment of hypercholesterolemia [15-17]. Up to now, there are still a lot of researches focusing on the development of novel inhibitors of HMG-CoA reductase [16,17]. Recently, a novel series of HMG-CoA reductase inhibitors, which can selectively inhibit HMG-CoA reductase with high inhibitory activities, has been reported by Pfefferkorn et al. [18].

Keeping the therapeutic significance of this class of inhibitors in mind and our continuing interest in the development of HMG-CoA reductase inhibitors, a 3D quantitative structure–activity relationship analysis (3D-QSAR) was proposed on the series of N-iso-propyl pyrrole-based derivatives reported for HMG-CoA reductase inhibitory activity [18]. The compounds reported were used for the purpose of 3D-QSAR modeling since they have a common scaffold and the biological assays have been carried out in the same lab.

3D-QSAR is widely used in the lead optimization stage and provides valuable insights about ligand–protein interactions [19,20]. In order to establish relationship between the spatial 3D pharmacophoric features and HMG-CoA reductase inhibitors activity of a group of N-iso-propyl pyrrole-based derivatives, a 3D-QSAR analysis was carried out on the combined dataset employing PHASE module of Schrodinger molecular modeling software [21].

In this study, we have developed a quantitative pharmacophore model based on HMG-CoA reductase inhibitors collected from the same laboratory [18]. The best quantitative model was used as a 3D search query for screening the ZINC database to identify new inhibitors of HMG-CoA reductase. Once identified, the candidate compounds were subsequently subjected to filtration by molecular docking.

## Methods

### Dataset

A set of 43N-iso-propyl pyrrole-based derivatives with well-defined HMG-CoA reductase inhibitory activity was used for the QSAR analysis [18]. In vitro inhibitory concentrations (IC_50_) of the molecules against HMG-CoA reductase were converted into corresponding pIC_50_ [−log(IC_50_)] and were used as dependent variables in the QSAR calculations (Additional file 1).

### Molecular modeling

PHASE 3.0 module of Schrodinger molecular modeling software was used to generate pharmacophore models [21]. In QSAR studies, appropriate conformation (lowest energy) of the compound is required for accurate calculation of 3D descriptors. All the 43 compounds were sketched by Maestro. Further, geometry optimization was carried out using the semi-empirical OPLS_2005 force field. All the molecules were divided into a training set and test set to maintain the structure and activity diversity in both the sets for QSAR model and pharmacophore generation and validation.

PHASE incorporates a structure cleaning step utilizing LigPrep, which attaches hydrogens, converts 2D structures to 3D, generates stereoisomers and neutralizes charged structures or determines the most probable ionization state at a user-defined pH. It also allows for the importation of 3D structures prepared outside its own workflow. Because one does not generally know the structure that a given molecule will adopt if and when it binds to a target protein, it is customary to represent each molecule as a series of 3D structures that sample the thermally accessible conformational states. For the purposes of pharmacophore model development, PHASE provides two built-in approaches, both of which employ the MacroModel conformational search engine. Since most of the studied ligands were flexible, all possible conformations for a ligand were generated to consider a range of conformations in order to increase the chances of finding the most active conformer close to the bound structure. Accordingly, the prepared ligands were subjected to conformational analysis using MonteCarlo Multiple Minimum method implemented in the Schrodinger software.

The ligands were assigned as active and inactive by giving an appropriate activity threshold value. The threshold value was 6 for active legend and 5 for inactive ligand**.** The activity threshold value was selected on the basis of dataset activity distribution (4.427–6.699) and the active ligands were chosen to derive a set of suitable pharmacophores. The so-prepared ligands were used for generating common pharmacophore and QSAR model building.

### Creation of pharmacophore sites

The chemical features of all ligands were defined by six pharmacophorics features: H-bond acceptor (A), H-bond donor (D), hydrophobic group (H), negatively charged group (N), positively charged group (P), and aromatic ring (R). An active analog approach was used to identify common pharmacophore hypotheses (CPHs), in which common pharmacophores were culled from the conformations of the set of active ligands using a tree-based partitioning technique that groups together similar pharmacophores according to their intersite distances [21].

### Scoring pharmacophores with respect to active and inactive ligands

The resulting pharmacophores were then scored and ranked. The scoring was done to identify the best candidate hypothesis, which provided an overall ranking of all the hypotheses. The scoring algorithm included the contributions from the alignment of site points and vectors, volume overlap, selectivity, number of ligands matched, relative conformational energy, and activity [22].

### Perceiving common pharmacophores

**Figure 1:**
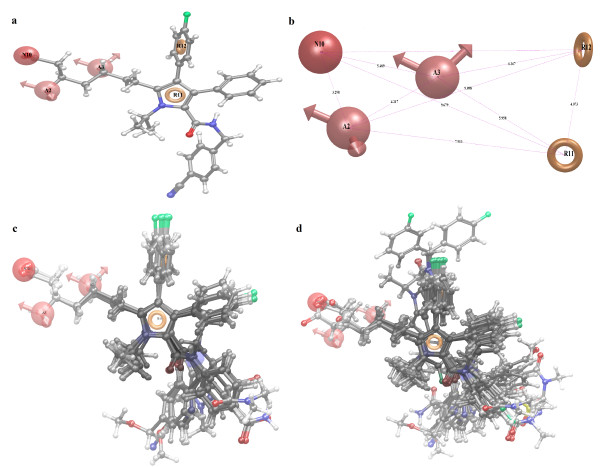
**Common pharmacophore generation and validation: (a)** Common pharmacophore aligned with most active ligand [two aromatic rings (dark yellow circle)], two acceptor [pink sphere with two arrows], and one negative ionic [pink sphere]; **(b)** common pharmacophoric sites of active ligand with distance. All distances are in Å unit; **(c)** alignment of all active ligands to the pharmacophore; and **(d) **alignment of all ligands (active and inactive) to the pharmacophore.

After the careful analysis of the scores and alignment of the active ligands to the generated hypothesis, a best pharmacophore hypothesis AANRR (Figure 1a) was selected for further studies (Table 1). The selected 3D pharmacophore hypothesis (Figure 1b) encompassed the following features: Two hydrogen bond acceptor (A) (pink sphere with two arrows), one negatively charged group (N) (pink sphere), two aromatic rings (R) (grey circle). The 2D representation (Figure 1a) of the pharmacophore AANRR shows the two hydroxyl groups (pink sphere with two arrows) hydrogen bond acceptor (A), carboxyl group (pink sphere) negatively charged group (N), pyrrole and benzene side chain rings viz*.* R11 and R12 are as the key pharmacophoric elements present in the selected pharmacophore.

**Table 1:** Score of different parameters of the AANRR hypothesis

**ID**	**Survival**	**Survival-inactive**	**Site**	**Vector**	**Volume**	**Selectivity**	**Matches**	**Energy**	**Activity**	**Inactive**
AANRR	3.603	1.593	0.9	0.967	0.732	2.077	12	4.741	6.097	2.011

### Building 3D-QSAR models

QSAR modeling was carried out using the selected hypothesis by dividing the dataset in to training set (70%) and test set (30%) in a random manner. PHASE presents two options for alignment of 3D structure of molecules: the pharmacophore based alignment and the atom-based alignment [21,23]. In this study, we have used an atom-based QSAR model, which is more useful in explaining the structure–activity relationship. In atom-based QSAR, a molecule is treated as a set of overlapping van der Waal’s spheres. Each atom (and hence each sphere) is placed into one of six categories according to a simple set of rules: hydrogens attached to polar atoms are classified as hydrogen bond donors (D); carbons, halogens, and C–H hydrogens are classified as hydrophobic/non-polar (H); atoms with an explicit negative ionic charge are classified as negative ionic (N); atoms with an explicit positive ionic charge are classified as positive ionic (P); non-ionic nitrogen and oxygen are classified as electron-withdrawing (W); and all other types of atoms are classified as miscellaneous (X).

For the purpose of QSAR development, van der Waal’s models of the aligned training set molecules were placed in a regular grid of cubes, with each cube allotted zero or more ‘bits’ to account for different types of atoms in the training set that occupy the cube. This representation gives rise to binary-valued occupation patterns that can be used as independent variables to create partial least-squares (PLS) QSAR models. Atom-based QSAR models were generated for the selected hypothesis using the 31-member training set using a grid spacing of 1.0 Å. The best QSAR model was validated by predicting activities of the 12 test set compounds. A four-component (PLS factor) model with good statistics was obtained for the dataset whereas the maximum number of PLS factors in each model can be 1/5 of the total number of training set molecules. Further increase in the number of PLS factors did not improve the model statistics or predictive ability [19].

### Protein preparation

The HMG-CoA reductase (PDB ID: 2R4F) was prepared using the Protein Preparation Wizard of the Schrödinger suite. H-atoms were added to the protein, including the protons necessary to define the correct ionization and tautomeric states of amino acid residues such as Asp, Ser, Glu, Arg, and His. The missing side chains of residues were corrected using Build interface incorporated in Maestro. For each structure, minimization was carried out with the Impact Refinement module, using the OPLS-2005 force field to alleviate steric clashes that may exist in the structures. The minimization was terminated when the energy converged or the Root Mean Square Deviation (RMSD) reached a maximum cutoff of 0.30 Å [24]. After protein preparation, receptor grid was set up, which was generated by employing the Receptor Grid Generation panel. Since this protein was associated with ligand, the ligand was selected to define the position and size of the active site.

### Virtual screening

AANRR hypothesis was used to search a 3D database for structures that match the pharmacophoric features of the model. Virtual screening was carried out using ZincPharmer that uses the pharmacophore to efficiently search the ZINC database of fixed conformers for pharmacophore matches [25,26]. To accomplish the best 3D similarity search, we used constraints that included maximum of 0.7 RMSD, obeying 10 rotatable bond cutoff and molecular weight range of 180–500 Dalton. A molecule which fits well with the pharmacophoric features of the AANRR hypothesis was retrieved as a hit.

### Docking methodology

All the hits with good RMSD value were enlisted and were incorporated to Maestro. Hits were subjected to hydrogen additions, removal of salt, ionization and generation of low-energy ring conformations using LigPrep. The chiralities of the original compounds were preserved. Finally, the low-energy 3D structures of all compounds were produced. The virtual screening workflow (VSW) in Maestro was used to dock and to score the lead-like compounds. In the first step, Glide was run in high-throughput virtual screen mode. 10% of the top-scoring ligands were kept to go onto the next, Glide Single Precision (SP), stage. The top-scoring 10% leads from GlideSP were retained and docked with Glide XP. All the Glide protocols were run using default parameters. An extensive search was carried out for generating all possible conformations. Minimization cycle for conjugate gradient and steepest descent minimizations were used with default value of 0.05 Å for initial step size and 1.0 Å for maximum step size. In convergence criteria for the minimization, both the energy change criteria and gradient criteria were used with default values of 10^-7^ and 0.001 kcal/mol, respectively. Then, all conformations were considered for docking. During the docking process, the Glide-score was used to select the best conformation for each ligand.

## Results and discussion

The aim of this study was to elucidate the 3D structural features of N-iso-propyl pyrrole-based derivatives (Table 2) crucial for binding, by generating 3D pharmacophore and to quantify the structural features of HMG-CoA reductase inhibitors essential for biological activity by generating atom-based 3D QSAR model. For the pharmacophore modeling and QSAR studies, we have used Phase module of Schrodinger suite. This hypothesis generated by Phase will also convey the relative binding of the ligands inside the active site of the receptor. Hence, we have used conformation suggested by the hypothesis for generating 3D-QSAR model to identify overall aspects of molecular structure that govern the activity.

**Table 2:** PLS statistical parameters of the selected 3D-QSAR model

**ID**	**PLS factors**	**SD**	***R***^**2**^	**F**	**P**	**RMSE**	***Q***^**2**^	**Pearson-R**
AANRR	1	0.4323	0.5171	31	5.181e-006	0.4018	0.3971	0.6346
	2	0.3191	0.7459	41.1	4.687e-009	0.4157	0.3549	0.676
	3	0.2369	0.865	57.7	7.249e-012	0.3565	0.5255	0.7782
	4	0.1369	0.9566	143.2	2.609e-017	0.2965	0.6719	0.8371

SD, standard deviation of the regression; *R*, squared value of *R*^2^ for the regression; *F*, variance ratio. Large values of *F* indicate a more statistically significant regression, *P*, significance level of variance ratio. Smaller values indicate a greater degree of confidence; RMSE,root-mean-square error, *Q*, squared value of *Q*^2^ for the predicted activities**,** Pearson-*R*, Pearson *R* value for the correlation between the predicted and observed activity for the test set.

For the generation of pharmacophore model, we have considered 12 compounds having activity > 6 against HMG-CoA reductase as active as they contain important structural features crucial for binding to the receptors binding site. We used four minimum sites and five maximum sites to have optimum combination of sites or features common to the most active compounds. One hundred and two common pharmacophore models were generated with different combination of variants in which all models were considered for further QSAR generation; the results were illustrated in (Additional file 2). Among these pharmacophores, the models which are showing the superior alignment with active compounds were identified by mapping to them and calculating the survival score. The survival scoring function identifies the best candidate hypothesis from the generated models and provides an overall ranking of all the hypotheses. The scoring algorithm includes contributions from the alignment of site points and vectors, volume overlap, selectivity, number of ligands matched, relative conformational energy, and activity. However, these pharmacophore models should also discriminate between the active (most active) and inactive (less active) molecules (Table 1). It is true that the hypothesis is incomplete if it lacks either a critical site that explains the binding or information on what prevents inactive ligands from binding. To identify the pharmacophore models with more active and less inactive features among these models, they were mapped to inactive compounds and scored. If inactive ligands score well, the hypothesis could be invalid because it does not discriminate between active and inactive ligands. Therefore, adjusted survival score was calculated by subtracting the inactive score from survival score of these pharmacophores (Additional file 2). Finally the models with maximum adjusted survival score and lowest relative conformational energy ware selected for generating pharmacophore (atom)-based alignment of HMG-CoA reductase inhibitors and model AANRR has been chosen because it produced good predictive power above other models. The special arrangement of features along with their distance present in five-featured pharmacophore, AANRR, was shown in Figure 1a. As depicted in the figure, among the two ring aromatic features, one feature is mapped to the pyrrole ring of all 12 active inhibitors and another on benzene side chain attached to pyrrole ring. The both hydrogen bond acceptor and negative ionic features are mapped to (the hydroxyl groups and carboxyl group) on N-iso-propyl side chain substituted on pyrrole ring.

**Figure 2:**
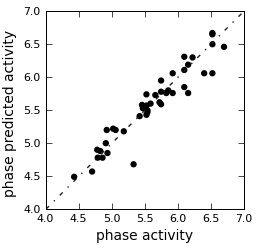
Fitness graph between observed activity versus phase-predicted activity for training and test set compounds.

For generating an atom-based 3D QSAR hypothesis, we have used a dataset of 31 (training set) compounds having inhibitory activity against HMG-CoA reductase. The model was validated using 12 (test set) compounds, which cover wide range of HMG-CoA reductase inhibitory activity. The alignment generated by the best pharmacophore model AANRR was used for QSAR model generation (Figure 1). Figure 1c presents good alignment of the active ligands and scattered alignment of inactive ligands to the developed pharmacophore model. Alignments of actives and of all inhibitors (active and inactive) are shown in Figure 1c,d**,** respectively. From Figure 1 we can easily identify that active ligand is having good alignment than inactive one. A four-PLS factor model with good statistics and predictive ability was generated for the dataset (Table 2). The number of PLS factor included in model development is four as incremental increase in the statistical significance and predictivity was observed for each incremental increase in the incorporated PLS factors up to four. The model express 95% variance exhibited by N-iso-propyl pyrrole-based derivatives, which is near to one and signifying a close agreement of fitting points on the regression line for the observed and PHASE-predicted activity that can be visualized from Figure 2 and is summarized in Table 3.

**Table 3:** Fitness and predicted activity data for test and training set of compounds

**Serial numbers**	**Ligand name**	**QSAR set**	**Activity**	**PLS factors**	**Predicted activity**	**Pharm set**	**Fitness**
1	228583	Training	4.427	4	4.49	Inactive	1.61
2	228954	Training	4.932	4	4.85	Inactive	1.6
3	228955	Training	6.523	4	6.67	Active	2.33
4	249273	Training	5.469	4	5.53		2.85
5	249724	Training	6.097	4	6.31	Active	2.85
6	249884	Training	4.921	4	5.2	Inactive	2.85
7	250088	Training	5.18	4	5.18		1.58
8	250090	Training	4.699	4	4.57	Inactive	1.57
9	250317	Training	6.523	4	6.5	Active	2.32
10	250500	Training	4.824	4	4.88	Inactive	2.36
11	250707	Training	5.42	4	5.41		2.79
12	250749	Training	6.155	4	6.19	Active	2.79
13	250953	Training	5.538	4	5.5		2.29
14	251499	Training	5.921	4	5.76		2.67
15	389216	Training	5.538	4	5.47		2.53
16	389217	Training	4.785	4	4.78	Inactive	2.2
17	389442	Training	5.523	4	5.57		2.55
18	391002	Training	5.523	4	5.43		2.62
19	394937	Training	5.745	4	5.78		2.8
20	398239	Training	5.824	4	5.76		2.87
21	398551	Training	6.222	4	6.3	Active	2.37
22	399313	Training	6.097	4	6.11	Active	2.98
23	399315	Training	5.745	4	5.59		2.19
24	399360	Training	4.907	4	5	Inactive	1.51
25	399771	Training	5.056	4	5.2		2.86
26	400560	Training	5.585	4	5.6		2.89
27	400874	Training	6.699	4	6.46	Active	2.89
28	400973	Training	6.398	4	6.06	Active	2.75
29	401293	Training	5.658	4	5.73		1.5
30	403127	Training	5.854	4	5.8		2.3
31	437774	Training	6.523	4	6.65	Active	2.3
32	228528	Test	5.721	4	5.62		2.53
33	228667	Test	5.018	4	5.22		1.64
34	249906	Test	5.921	4	6.06		2.9
35	387514	Test	5.456	4	5.58		2.4
36	389002	Test	5.745	4	5.95		2.34
37	389441	Test	4.857	4	4.78	Inactive	2.19
38	389443	Test	5.328	4	4.68		1.62
39	398240	Test	6.097	4	5.85	Active	3
40	399773	Test	6.523	4	6.06	Active	2.29
41	400747	Test	6.155	4	5.76	Active	2.78
42	438662	Test	4.777	4	4.9	Inactive	2.22
43	438694	Test	5.523	4	5.74		2.39

A 3D-QSAR analysis was performed on the series of N-iso-propyl pyrrole-based derivatives to understand the effect of spatial arrangement of structural features on their HMG-CoA reductase inhibition. The large value of F (143.2) indicates a statistically significant regression model, which is also supported by the small value of the variance ratio (P), an indication of a high degree of confidence. Further, small values of standard deviation (0.137) of the regression and RMSE (RMSE = 0.296) makes an obvious implication that the data used for model generation are best for the QSAR analysis. Validity of the model can be expressed by cross-validated correlation coefficient (*q*^2^ = 0.672) that was obtained by leave one out or leave *N* out method. The *q*^2^ is more reliable and robust statistical parameter than *r*^2^ because it is obtained by external validation method by dividing the dataset into training and test set.

**Figure 3:**
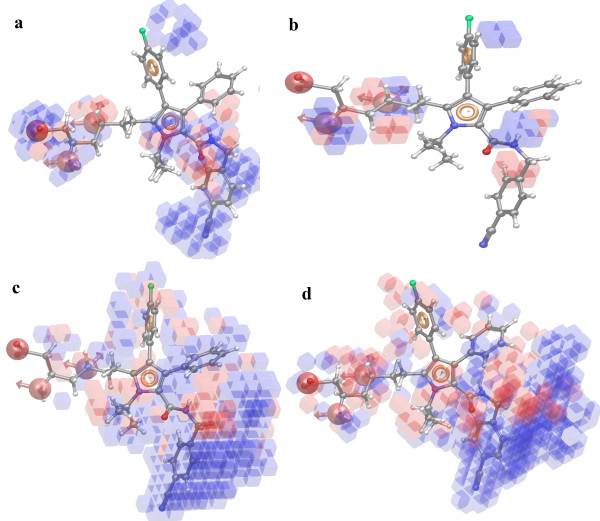
**QSAR visualization of various substituents effect: (a)** electron withdrawing feature; **(b)** hydrogen-bond donor; **(c)** hydrophobic features; and **(d)** combined effect (blue cubes showing positive potential while red cubes showing negative potential of particular substitution).

The atoms visualize 3D characteristics of the ligands (atoms or pharmacophores) as that contribute positively or negatively to activity. The QSAR model displays 3D characteristics (Figure 3) as cubes that represent the model and color according to the sign of their coefficient values, which by default is blue for positive coefficients and red for negative coefficients. Positive coefficients indicate an increase in activity, negative coefficients a decrease. The visualization of the coefficients is useful to identify characteristics of ligand structures that tend to increase or to decrease the activity. This might give a clue to what functional groups are desirable or undesirable at certain positions in a molecule. The blue cubes in 3D plots of the 3D pharmacophore regions refer to ligand regions in which the specific feature is important for better activity, whereas the red cubes demonstrates that particular structural feature or functional group, which is not essential for the activity or likely the reason for decreased binding potency.

Visual analysis of Figure 3a demonstrates that the presence of the blue cubes at the A2, A3, N10 (N-iso-propyl side chain), cyano group, and amide group (carbamoyl side chain) attached to pyrrole ring is pointing out the positive potential of electron withdrawing characteristic of the molecules and is requisite for the activity at this particular place. It can be suggested that addition of appropriate electron withdrawing groups at the A2, A3, cyano, and amide (carbamoyl side chain) sites will append the HMG-CoA reductase inhibition, whereas the addition of electron-withdrawing groups near carboxy group attached to pyrrole ring position will contribute to decreased receptor binding, which in turn will result in lower potency of compounds because red color cubes are for negative effect of electron-withdrawing group to that particular place. Figure 3b illustrates that H-bond donor characteristic is necessary at A2 and A3 (N-iso-propyl side chain) present on the pyrrole ring. The red cubes at the benzene ring attached to the amide side chain of pyrrole ring demonstrate a negative potential of H-bond donor at that position on the compounds. Figure 3c demonstrates the effect of hydrophobic groups on HMG-CoA reductase inhibition. It can be deduced from the figure that hydrophobic groups are well tolerated near cyano benzene ring on carbamoyl side chain (blue cubes), while the substitution of hydrophobic groups in between amide (carbamoyl side chain) and cyano benzene ring site are unacceptable (red cubes) or may hinder the binding of the molecules to the receptor active site and will result in decreased HMG-CoA reductase inhibition. Further, Figure 3d showed combined effect of all the features. The presence of hydroxyl groups (N-iso-propyl side chain), cyano group, and amide group (carbamoyl side chain) will have the positive effect (blue cubes).

### Virtual screening

One efficient approach to drug discovery is the virtual screening of the molecular libraries. Pharmacophore-based database searching is considered as a type of ligand-based virtual screening, which can efficiently be used to find novel and potential leads for further development. A potent pharmacophore model possesses the chemical functionalities responsible for bioactivities of potential drugs; therefore, it can be used to perform a database search by serving as a 3D query. The pharmacophore AANRR was used as a 3D structural query for retrieving potent molecules from the ZINC chemical database through ZincPharmer. To avoid possible incorrect predictions, the molecular weight cutoff of 180–500 Dalton range, RMSD of 0.7, and cutoff limit of 10 rotatable bonds filters were applied. Altogether, 3D structural query retrieved 17,283 compounds from the ZINC database.

### Drug likeness analysis

The ZINC database was evaluated for drug-likeness of the lead molecules by assessing their physicochemical properties and by applying Lipinski’s rule of five. Their molecular weights were <500 Daltons with <5H-bond donors, <10H-bond acceptors and a log p of <5; these properties were well within the acceptable range of the Lipinski rule for drug-like molecules.

These compounds were further evaluated for their drug-like behavior through the analysis of pharmacokinetic parameters required for absorption, distribution, metabolism, and excretion (ADME). For the four lead compounds, the partition coefficient (QPlogPo/w) and water solubility (QPlogS), critical for estimation of absorption and distribution of drugs within the body, ranged between −2 to 6.5 and −6 to −0.5, respectively. Further, the blood brain barrier permeability, which is prerequisite for the entry of drugs to the brain, was found to be in the acceptable range (−3 to 1.2). Overall, the percentage human oral absorption of all four compounds ranged from 30 to 79%. All these pharmacokinetic parameters were within the acceptable range defined for human use, thereby indicating their potential as drug-like molecules (Table 4).

**Table 4:** ADME properties of selected hits with the docking score

**ZINC ID**	**log*Po*/*w***^**a**^	**log*S***^**b**^	**logHERG**^**c**^	**logBB**^**d**^	**% Oral absorption**^**e**^	**Predicted activity**	**Docking score**
14010678	3.61	−5.58	−3.61	−1.97	57.81	5.7	−9.22
35655503	2.05	−4.10	−2.87	−1.04	70.66	5.52	−9.34
26508465	−0.97	−2.25	−3.06	−2.22	32.50	5.98	−9.16
02554357	3.45	−6.05	−3.93	−0.92	79.57	5.23	−8.95

^a^Predicted octanol/water partition co-efficient log *p* (acceptable range: −2.0 to 6.5).

^b^Predicted aqueous solubility; *S* in mol/L (acceptable range: −6.5 to 0.5).

^c^Predicted IC_50_ value for blockage of HERG K+ channels (acceptable range: below −6.0).

^d^Predicted blood brain barrier permeability(acceptable range: −3 to 1.2).

^e^Percentage of human oral absorption (.25% is poor and .80% is high).

### Molecular docking

Docking was carried out to increase the power of the pharmacophore-based screening to discriminate between the active and inactive ligands (that could adopt poses to accomplish the pharmacophore features). The docking was especially useful during the second step of the VS workflow for the identification of molecules having at least one conformer that irrespective of its initial spatial orientation, can fit into the pharmacophore (because docking can be used to exclude those inactive molecules from the VS having at least one conformer, which matches the sites in the pharmacophore but is sterically hindered by other residues in the active site).

**Figure 4:**
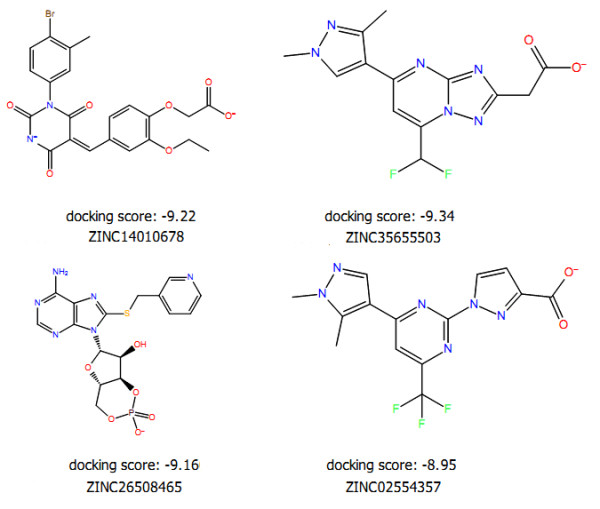
The chemical structures of final hits represented in 2D with docking scores.

To further refine the retrieved candidates, the 17,283 candidates were docked (VSW) into the active site of HMG-CoA reductase and screened through developed QSAR model in order to predict the activities. The binding modes for these compounds identified by docking were ranked according to the information obtained by different scoring constraints. Based on the knowledge of existing HMG-CoA reductase inhibitors, ADME properties, docking score, and the active site requirements, we selected four compounds from highest scoring structure. The four candidates were shown to exhibit highest binding affinities and the predicted activity (Table 4). The diversity of the candidates demonstrated that the pharmacophore model was able to retrieve candidates with similar features to the existing HMG-CoA reductase inhibitors as well as novel scaffolds (Figure 4).

## Conclusion

A ligand-based pharmacophore model was generated for the series of N-iso-propyl pyrrole-based derivatives with HMG-CoA reductase inhibitory activity to reveal the structural features responsible for biological activity. With the help of pharmacophore-based alignment of HMG-CoA reductase inhibitors, a meaningful 3D-QSAR was derived to identify and how three-dimensional arrangements of various substituents will affect the HMG-CoA reductase inhibition. The selected model as shown by the correlation statistics and predictive statistics is very much significant to draw unambiguous inferences. Further, the generated 3D-QSAR model also explains how and at what extent electron withdrawing, hydrophobic and H-Donor moieties in the molecular structure influences HMG-CoA reductase inhibition showed by the title compounds. The model shows that HMG-CoA reductase inhibition can be increased, if the electron-withdrawing character near N-iso-propyl side chains is supplemented by appropriate functional groups and by the incorporation of H-donor, hydrophobic, and positive ionic groups at specific positions in the molecules. Finally, four potential hits with good fitness value and predicted activity were identified by virtual screening and docking, whose activity can be further improved with the help of this QSAR model. This study provides a set of guidelines which will greatly help in designing the newer and more potent HMG-CoA reductase inhibitors of the N-iso-propyl pyrrole-based scaffold.

## Competing interests

Both authors declare that they have no competing interests.

## Authors’ contributions

MKT designed methods and experiments, carried out the *in silico* experiments, analyzed the data, interpreted the results and wrote the paper. RGK was involved in the conception of the idea, drafting the manuscript or revising it critically and has given final approval of the version to be published. All authors have contributed to, seen and approved the manuscript.

## Supplementary Material

Additional file 1 Inhibition data of N-iso-propyl pyrrole-based derivatives. Click here for file

Additional file 2 Selected pharmacophore models with good survival score. Click here for file
