# Supplementary material for: Pharmacophore generation and atom-based 3D-QSAR of N-iso-propyl pyrrole-based derivatives as HMG-CoA reductase inhibitors
Source: Org Med Chem Lett. 2012 Jul 2;2:25. doi: 10.1186/2191-2858-2-25 (PMC3519668; doi:10.1186/2191-2858-2-25)
Supplement: Additional file 1 — Inhibition data of N-iso-propyl pyrrole-based derivatives. [file 2191-2858-2-25-S1.doc]

**Table 1. Inhibition data of N-iso-propyl pyrrole-based derivatives**


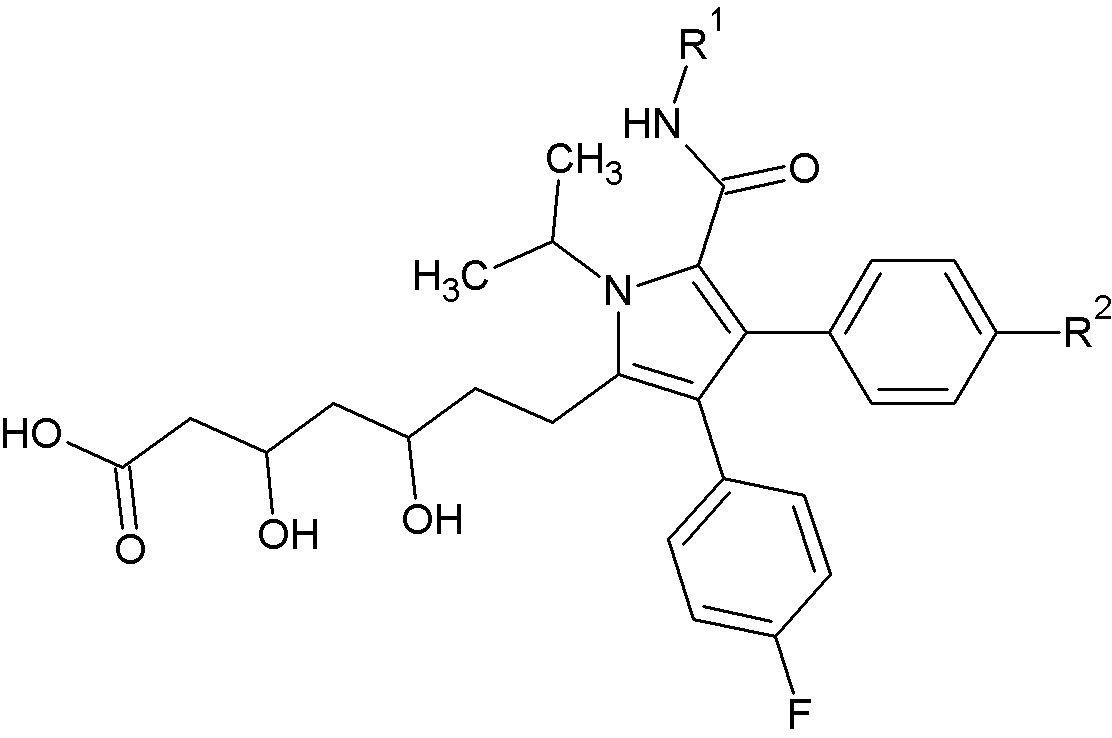


| **S.NO** | **CHEM ID** | **R1** | **R2** | **pIC50** |
| --- | --- | --- | --- | --- |
| 1 | 249273 | 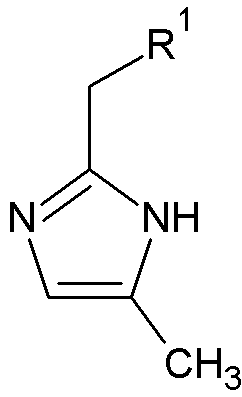 | -H | 5.469 |
| 2 | 249724 | 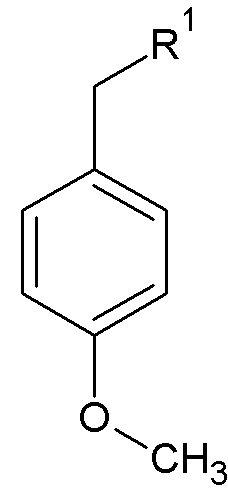 | -H | 6.097 |
| 3 | 249884 | -C2H5 | -H | 4.921 |
| 4 | 249906 | 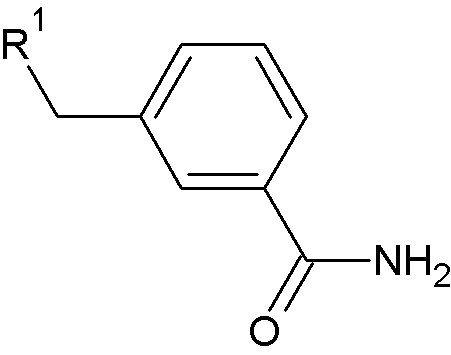 | -H | 5.921 |
| 5 | 250088 | 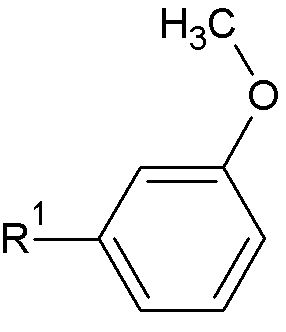 | -F | 5.18 |
| 6 | 250090 | -CH3, -CH3 | -H | 4.699 |
| 7 | 250317 | 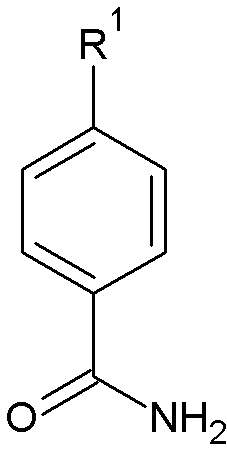 | -H | 6.523 |
| 8 | 250500 | 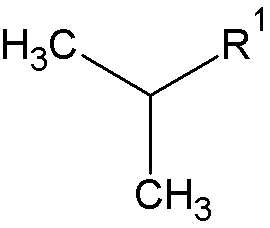 | -H | 4.824 |
| 9 | 250707 | 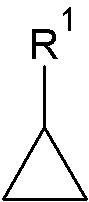 | -H | 5.42 |
| 10 | 250749 | 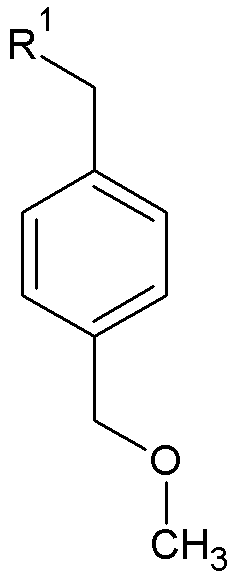 | -H | 6.155 |
| 11 | 250953 | 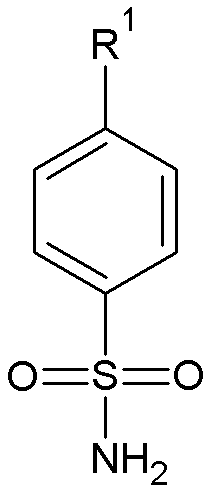 | -H | 5.538 |
| 12 | 251499 | 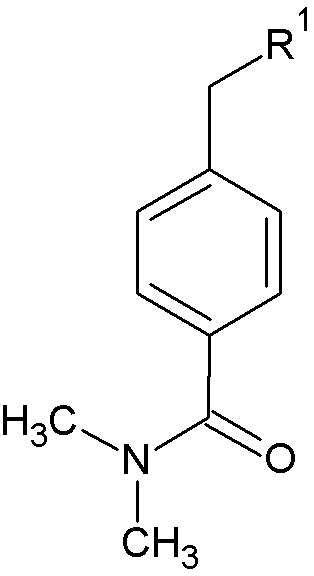 | -H | 5.921 |
| 13 | 389002 | 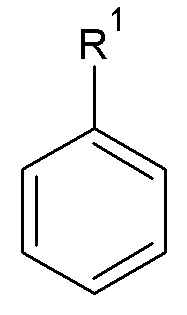 | -F | 5.745 |
| 14 | 391002 | 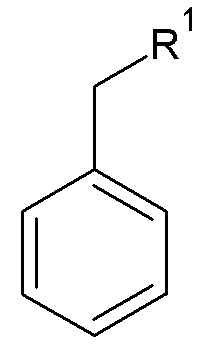 | -H | 5.523 |
| 15 | 394937 | 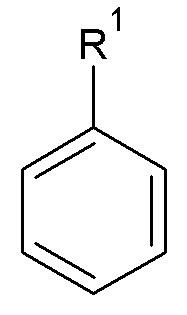 | -H | 5.745 |
| 16 | 398239 | 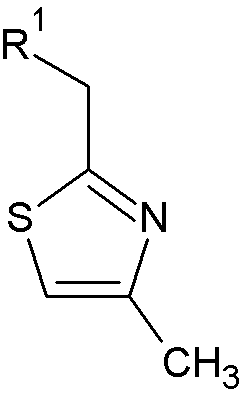 | -H | 5.824 |
| 17 | 398240 | 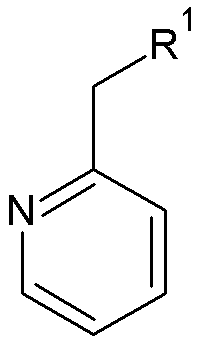 | -H | 6.097 |
| 18 | 398551 | 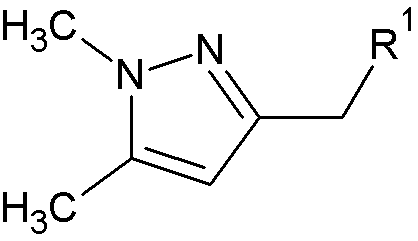 | -H | 6.222 |
| 19 | 399313 | 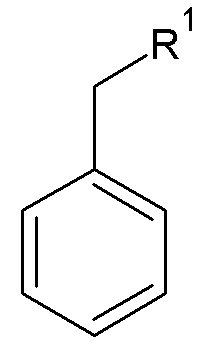 | -H | 6.097 |
| 20 | 399315 | 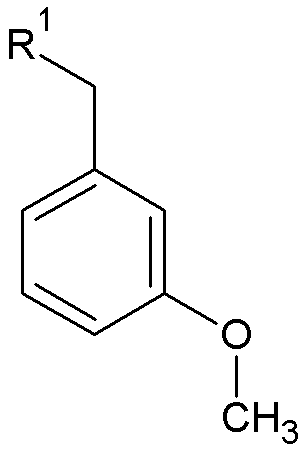 | -H | 5.745 |
| 21 | 399360 | 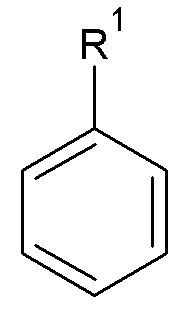 | -F | 4.907 |
| 22 | 399771 | -CH3 | -H | 5.056 |
| 23 | 399773 | 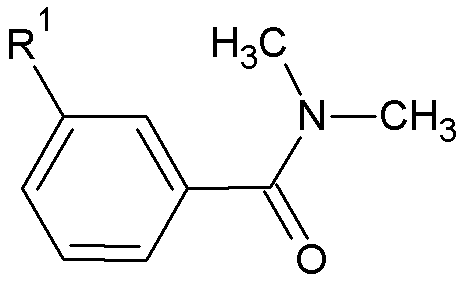 | -F | 6.523 |
| 24 | 400560 | 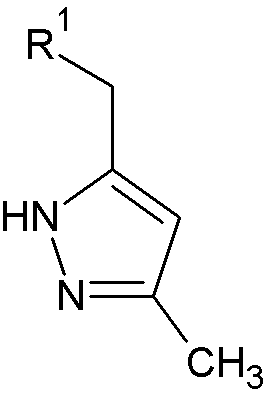 | -H | 5.585 |
| 25 | 400747 | 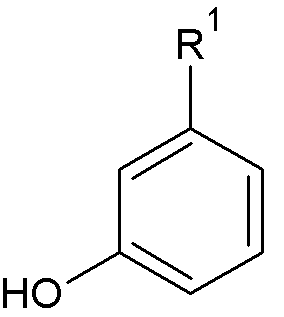 | -F | 6.155 |
| 26 | 400874 | 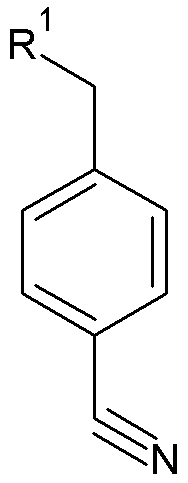 | -H | 6.699 |
| 27 | 400973 | 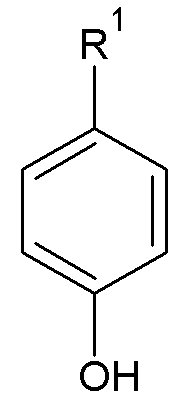 | -H | 6.398 |
| 28 | 401293 | 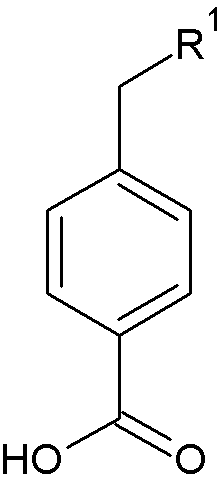 | -H | 5.658 |
| 29 | 403127 | 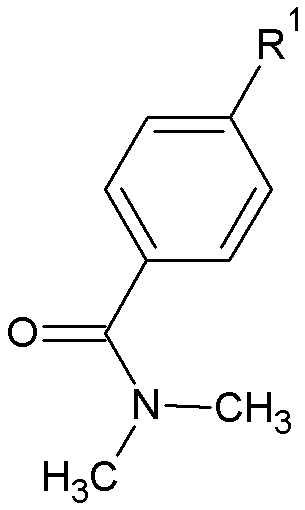 | -H | 5.854 |
| 30 | 437774 | 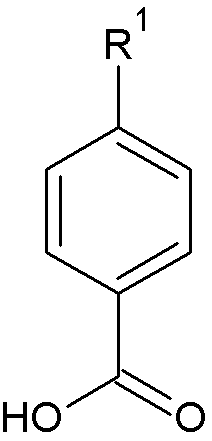 | -H | 6.523 |
| 31 | 438694 | -H | -H | 5.523 |


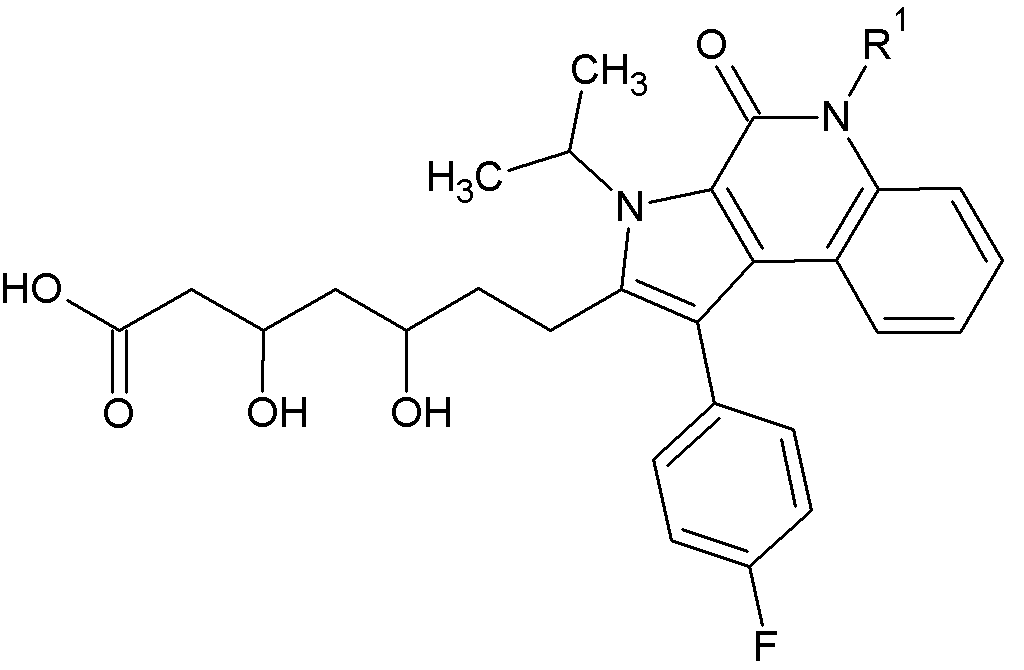


| **S.NO** | **CHEM ID** | **R1** | **pIC50** |
| --- | --- | --- | --- |
| 32 | 228528 | 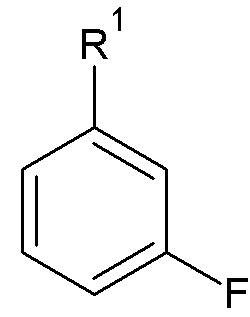 | 5.721 |
| 33 | 228583 | 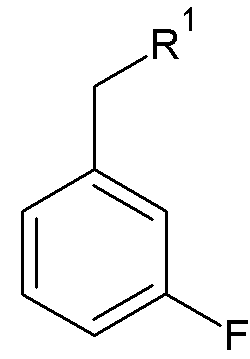 | 4.427 |
| 34 | 228667 | 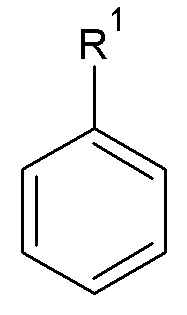 | 5.018 |
| 35 | 228954 | -H | 4.932 |
| 36 | 389216 | 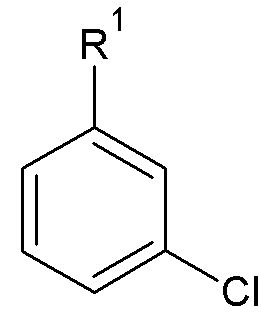 | 5.538 |
| 37 | 389217 | 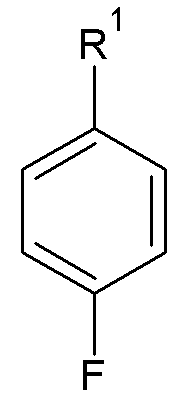 | 4.785 |
| 38 | 389441 | 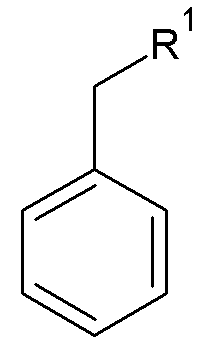 | 4.857 |
| 39 | 389442 | 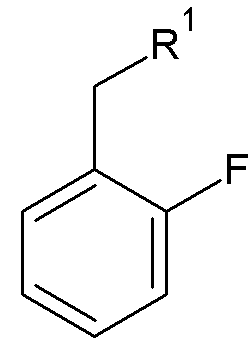 | 5.523 |
| 40 | 389443 | 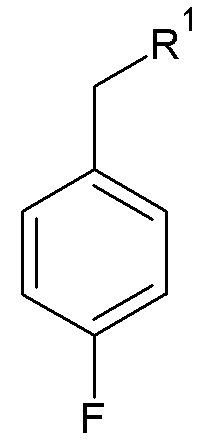 | 5.328 |
| 41 | 438662 | 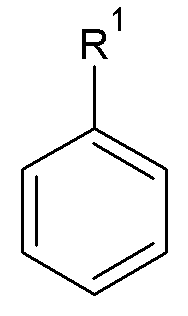 | 4.777 |


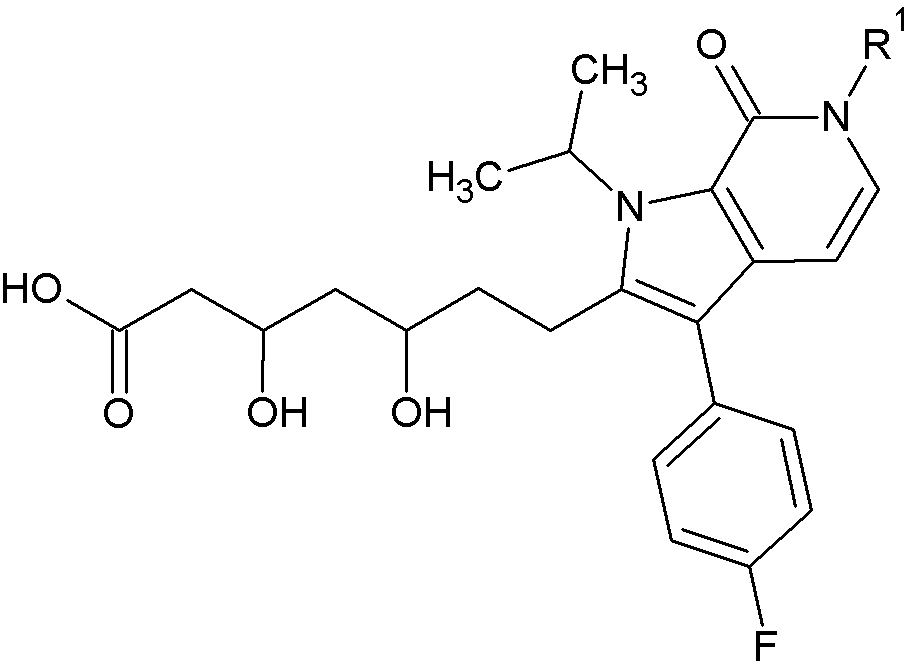


| **S.NO** | **CHEM ID** | **R1** | **pIC50** |
| --- | --- | --- | --- |
| 42 | 228955 | 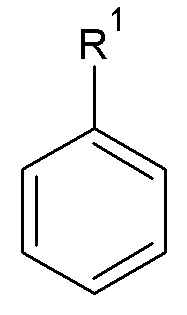 | 6.523 |
| 43 | 387514 | 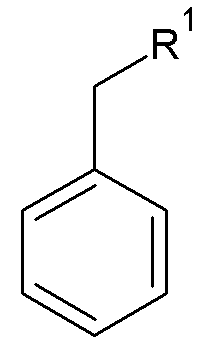 | 5.456 |
