# Supplementary material for: Pharmacophore generation and atom-based 3D-QSAR of N-iso-propyl pyrrole-based derivatives as HMG-CoA reductase inhibitors
Source: Org Med Chem Lett. 2012 Jul 2;2:25. doi: 10.1186/2191-2858-2-25 (PMC3519668; doi:10.1186/2191-2858-2-25)
Supplement: Additional file 2 — Selected pharmacophore models with good survival score. [file 2191-2858-2-25-S2.doc]

**Additional file 2:** Selected pharmacophore models with good survival score.

| **Sl No** | **ID** | **Survival** | **Survival -inactive** | **Adjusted Survival** | **Site** | **Vector** | **Volume** | **Selectivity** | **Matches** | **Energy** | **Activity** | **Inactive** |
| --- | --- | --- | --- | --- | --- | --- | --- | --- | --- | --- | --- | --- |
| 1 | AAARR.11091 | 3.61 | 1.246 | 2.364 | 0.91 | 0.987 | 0.714 | 1.508 | 12 | 5.549 | 6.523 | 2.364 |
| 2 | AAARR.12920 | 3.608 | 1.116 | 2.492 | 0.92 | 0.989 | 0.702 | 1.53 | 12 | 7.183 | 6.097 | 2.492 |
| 3 | AANRR.10061 | 3.608 | 1.102 | 2.506 | 0.89 | 0.978 | 0.74 | 2.169 | 12 | 2.231 | 6.097 | 2.506 |
| 4 | ADHNR.10054 | 3.605 | 1.292 | 2.313 | 0.92 | 0.985 | 0.7 | 2.249 | 12 | 2.958 | 6.097 | 2.313 |
| 5 | AAARR.10343 | 3.605 | 1.136 | 2.469 | 0.89 | 0.988 | 0.729 | 1.487 | 12 | 1.804 | 6.398 | 2.469 |
| **6** | **AANRR.10786** | **3.603** | **1.593** | **2.01** | **0.9** | **0.967** | **0.732** | **2.077** | **12** | **4.741** | **6.097** | **2.011** |
| 7 | AHNRR.10437 | 3.603 | 1.107 | 2.496 | 0.9 | 0.989 | 0.713 | 2.267 | 12 | 2.787 | 6.155 | 2.495 |
